# Supplementary material for: Multivariate GWAS of Alzheimer’s disease CSF biomarker profiles implies GRIN2D in synaptic functioning
Source: Genome Med. 2023 Oct 4;15:79. doi: 10.1186/s13073-023-01233-z (PMC10548686; doi:10.1186/s13073-023-01233-z)
Supplement: Supplementary file 2 — Additional file 2: Supplementary Methods and Figures. Additional methods on genotyping, multiple testing adjustment, replication study and figures S1-S5. [file 13073_2023_1233_MOESM2_ESM.pdf]

Supplementary Methods

X-chromosomal genotyping, imputation and quality control (QC)

For genotypes on the X chromosome, QC for non-pseudoautosomal regions markers was performed separately for male and female subjects. SNPs were excluded with MAF below 1% in the female group, genotyping rates below 98%, deviations from HWE in control individuals ($p<1\times10^{-4}$), differential genotyping efficiency between women and men ($p< 1\times10^{-4}$), differential allele frequency between women and men ($p< 1\times10^{-4}$) or ambiguous allele combinations (A/T and C/G). Markers in the pseudoautosomal regions (PAR1, PAR2) were processed analogous to the autosomes. We then aligned the alleles of the remaining SNPs to the reference genome "GRCh37/hg19" before imputation. Imputations of untyped X-Chromosomal markers (including PAR) were subsequently performed on the Sanger Institute imputation server (https://imputation.sanger.ac.uk/) using the extended HRC reference panel available at that site.[1] After imputation, we used the same QC criteria as for the autosomal SNPs on markers in PAR. For remaining non-PAR markers SNPs were excluded with MAF below 1% in male and female separately, imputation quality ($R^2<0.30$) and deviations from HWE ($p<5\times10^{-4}$).

Multiple testing adjustment

To strike a balance between reliable inference and power, we present our findings as primary, secondary and tertiary results. The primary analyses in this study were the GWAS in the full dataset independent of sex. For SNP-based tests we apply the conventional genome-wide association threshold of $p<5*10^{-8}$ and for gene-based tests we used Bonferroni's method to adjusted for 19,511 genes resulting in a

threshold of $p<2.3*10^{-6}$, as recommended by FUMA. The sex-specific analyses present additional tests of related (and non-independent) hypothesis, and, thus should be regarded as secondary and more exploratory analyses.

Next, we aimed to better understand the potential consequences of identified loci on latent AD and selected them for further mediation analyses. Here, we assume that the genome-wide significant SNPs are truly associated with a biomarker PC and test the hypothesis, that they are also associated with latent AD. As each SNP can be associated with latent AD either directly or via any of the five biomarker PCs, we adjusted for 6 possible pathways, resulting in a Bonferroni-adjusted alpha of $0.05/6=p<0.0083$.

In case of comparisons to rare variants, we tested SNPs based on prior evidence of rare variants associations. We therefore only adjusted for number of SNPs within the selected loci. To account for non-independence between SNPs, we adjusted for the number of effective tests, taking into account the eigenvalue of correlation between SNPs. We estimated the number of effective tests using the Li & Ji method[2], as implemented in poolR[3]. In the current study, we assessed 564 SNPs within *IFFO1*, *DTNB*, *NLRC3* and *SLC22A10* for association with the injury/inflammation component (PC3). The estimated number of effective tests was 127, resulting in a Bonferroni-adjusted alpha of $0.05/127=3.9*10^{-4}$. In case of *GABBR2* and *CASZ,* which were tested for association with non-AD synaptic functioning (PC5), we analyzed 1837 common genetic variants within both genes, 312 of which were independent resulting in a Bonferroni adjusted alpha of $p<0.05/312=1.6*10^{-4}$.

References

1. McCarthy, S., Das, S., Kretzschmar, W., Delaneau, O., Wood, A.R., Teumer, A., Kang, H.M., Fuchsberger, C., Danecek, P., Sharp, K., et al. (2016). A reference panel of 64,976 haplotypes for genotype imputation. Nat. Genet. *48*, 1279–1283.

2. Li, J., and Ji, L. (2005). Adjusting multiple testing in multilocus analyses using the eigenvalues of a correlation matrix. Heredity (Edinb). *95*, 221–227.

3. Cinar, O., and Viechtbauer, W. meff: Number of Effective Tests.

Knight Alzheimer Disease Research Center (Knight-ADRC) cohort

The Knight ADRC at Washington University School of Medicine (St. Louis, MO, USA) recruits and longitudinally assesses community-dwelling adults older than 45 years old via prospective studies of memory and aging since 1979. All studies were approved by the Human Research Protection Office at Washington University, and written informed consent was obtained from all participants. The Memory and Aging Project at the Knight ADRC (Knight ADRC-MAP) involves longitudinal collection of biofluids (plasma, CSF, fibroblast), annual clinical assessments, neuropsychological testing, and neuroimaging studies, as well as collection of autopsied brain samples. Eligible participants may be asymptomatic or have mild dementia at the time of enrollment. All participants are required to participate in core study procedures, including annual longitudinal clinical assessments, neuropsychological testing, neuroimaging, and biofluid biomarker studies. Annual assessments of the participants were performed by experienced clinicians using a semi-structured interview with knowledgeable collateral source and the symptomatic individual in accordance with the Uniform Data Set protocol of the National Alzheimer's Coordinating Center[1], as well as a detailed neurological examination. Participants comprise Non-Hispanic White individuals from North America (82.5%) and African-Americans (13.3%). Samples have been obtained from over 5,510 participants, including 2,426 AD cases, 148 FTD, 88 DLB, and 2,156 cognitively normal healthy individuals. Autopsy material are available for over 1,182 participants including 474 with fresh frozen parietal brain tissue (https://dss.niagads.org/datasets/ng00127/). Multi-tissue (brain, CSF, and plasma), multi-omics data (genetics, epigenomics, transcriptomics, proteomics, and metabolomics) have been generated for the purpose of identifying novel risk and

protective variants for dementia, and potential drug targets. Participants from the Knight ADRC were included in this study if they were cognitively unimpaired with a global clinical dementia rating (CDR) score of 0 at enrollment. A clinical diagnosis of incident dementia is considered by study clinicians at the conclusion of each annual assessment, integrating results from the clinical assessment and bedside measures of cognitive function.[2] Dementia was diagnosed according to the National Institute of Neurological Disorders and Stroke criteria[3] and National Institute on Aging-Alzheimer's Association Work Group criteria for participants assessed after 2011.[4] Diagnosis of AD dementia was made in accordance with criteria developed by working groups from the National Institute of Aging and the Alzheimer's Association.[4] Diagnosis of vascular dementia conformed to the NINDS-AIREN criteria.[5]

1. Morris JC, Weintraub S, Chui HC, et al. The uniform data set (UDS): clinical and cognitive variables and descriptive data from Alzheimer disease centers. *Alzheimer Dis Assoc Disord*. 2006;20:210-216

2. Day GS, Lim TS, Hassenstab J, et al. Differentiating cognitive impairment due to corticobasal degeneration and Alzheimer disease. *Neurology*. 2017;88(13):1273-1281

3. McKhann G, Drachman D, Folstein M, et al. Clinical diagnosis of Alzheimer's disease: report of the NINCDS-ADRDA Work Group under the auspices of Department of Health and Human Services Task Force on Alzheimer's Disease. *Neurology* 1984;34:939–944

4. McKhann GM, Knopman DS, Chertkow H, et al. The diagnosis of dementia due to Alzheimer's disease: recommendations from the National Institute on Aging-

Alzheimer's Association workgroups on diagnostic guidelines for Alzheimer's disease. *Alzheimers Dement* 2011;7:263–269

5. Roman GC, Sachdev P, Royall DR, et al. Vascular cognitive disorder: a new diagnostic category updating vascular cognitive impairment and vascular dementia. *J Neurol Sci*. 2004;226:81–7

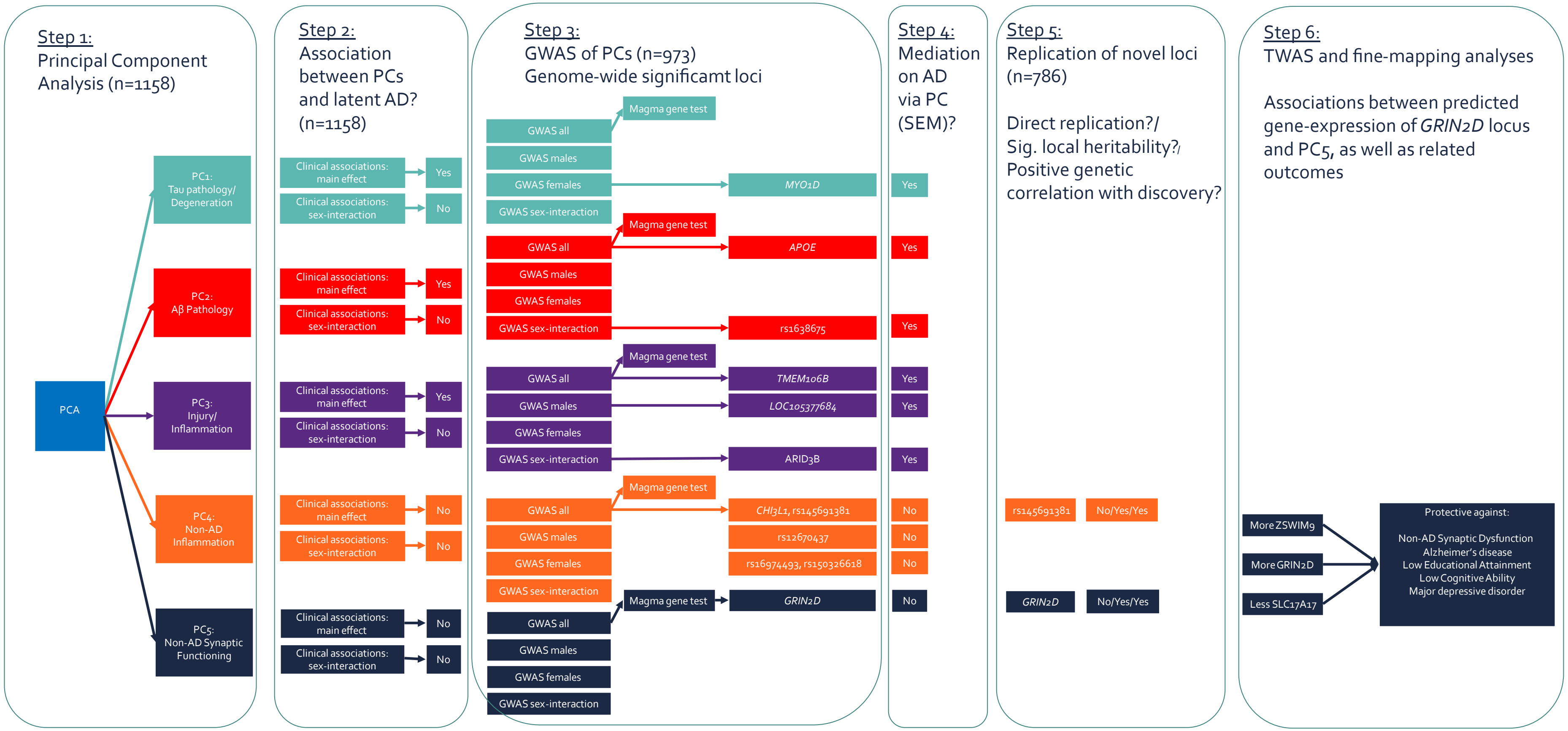

Fig. S1: Schematic work-flow of the analyses performed in our study

.

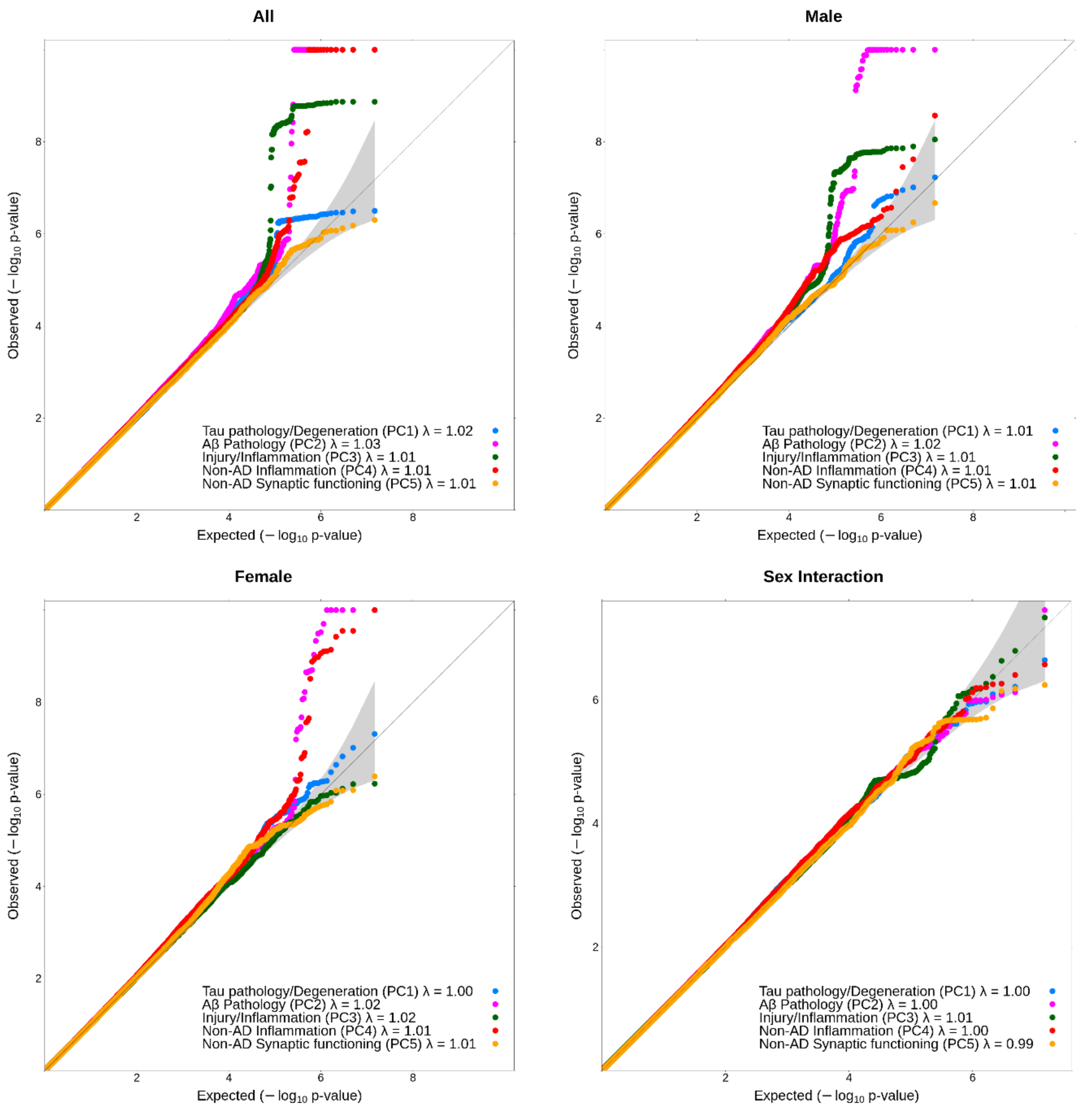

Fig. S2: QQ-plots of SNP test statistics. Diagonal line indicates a p-value distribution expected by chance. Dots represent p-values per SNP for each of the outcomes (see legend for color coding). Each panel depicts the p-value distribution for different GWAS models, either main effects (all sexes), male or female-stratified, or the p-values of a sex interaction term.

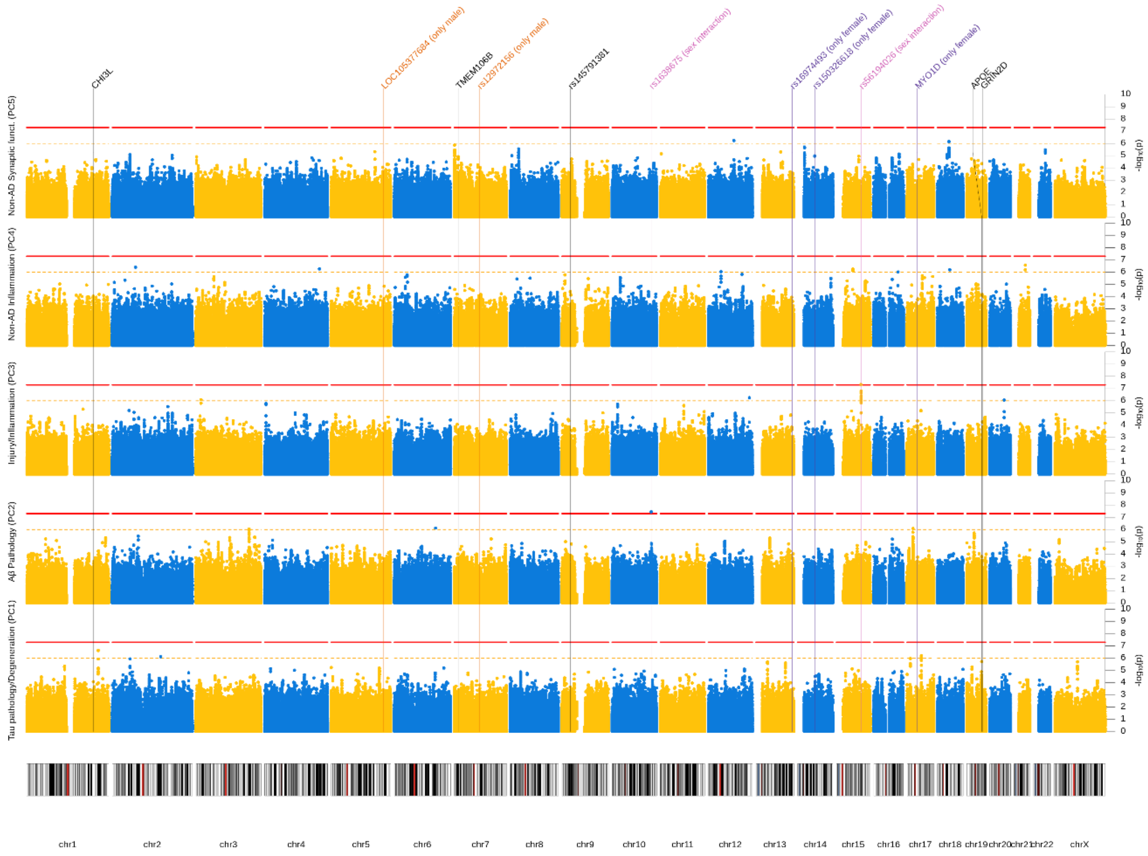

Fig. S3: Manhattan plot (sex interaction). Sex-interaction results from GWAS of five CSF biomarker PC. Each row represents a different PC as outcome. X-axis represents each SNP and the y-axis the p-value of the sex-interaction term on a -$\log_{10}$ scale. All analyses were adjusted for genetic ancestry and SNP array. Red line indicates genome-wide significance threshold ($p=5*10^{-8}$). Yellow line indicates suggestive threshold ($p=1*10^{-6}$). Vertical lines point towards genome-wide significant loci based on any model. P-values below $1*10^{-10}$ were winsorized to $1*10^{-10}$

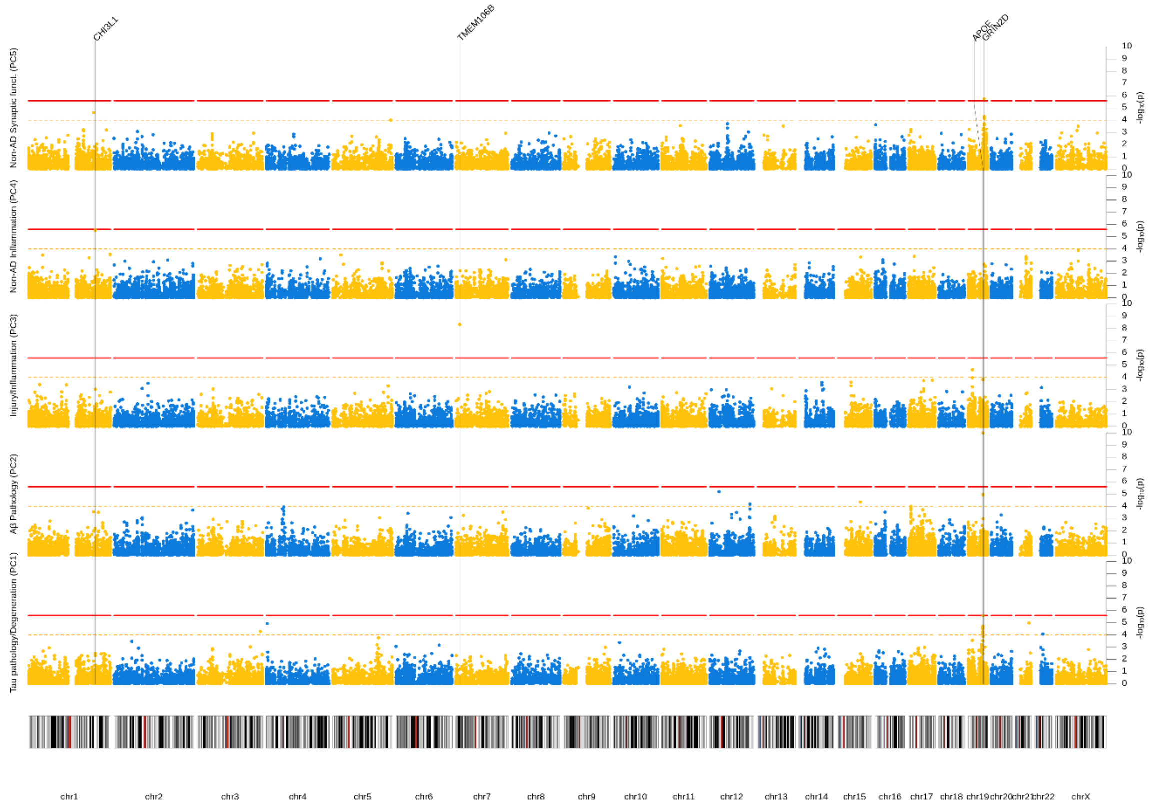

Fig. S4: Manhattan plot (gene-based tests). Results from GWAS of five CSF biomarker PC across both sexes. Each row represents a different PC as outcome. X-axis represents each SNP and the y-axis the p-value of the gene-based association derived with MAGMA on a $-\log_{10}$ scale. All analyses were adjusted for sex, genetic ancestry and SNP array. Red line indicates genome-wide significance threshold ($p=2.3*10^{-6}$). Yellow line indicates suggestive threshold ($p=1*10^{-4}$). Vertical lines point towards genome-wide significant loci based on any model. P-values below $1*10^{-10}$ were winsorized to $1*10^{-10}$.

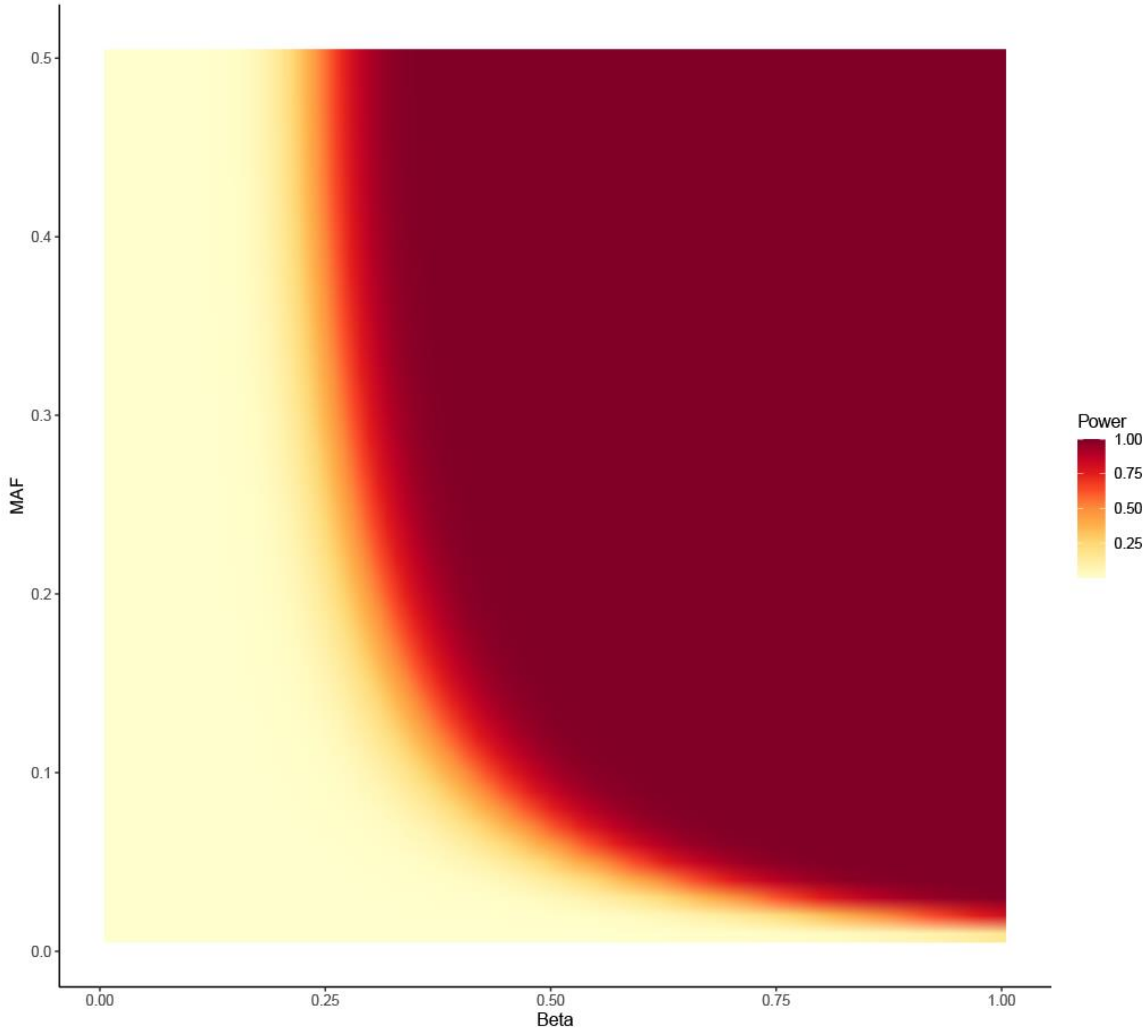

Fig. S5: Power-curve for GWAS main analyses (n=973). Power as function of effect size (beta) and minor allele frequency (MAF) for an alpha level representing genome-wide significance ($5*10^{-8}$). Created with gwas-power: https://github.com/kaustubhad/gwas-power
